# Companion-oriented AI chatbot use and loneliness among Chinese emerging adults: the mediating role of self-attributed offline social displacement and the moderating role of relational-interdependent self-construal

**DOI:** 10.3389/fpsyg.2026.1933530

**Published:** 2026-10-22

**Authors:** Taorong Xiao

**Affiliations:** School of Media and Communication, Yunnan Normal University, Kunming, China

**Keywords:** companion AI chatbot, emerging adulthood, loneliness, relational-interdependent self-construal, self-attributed offline social displacement

## Abstract

**Objective:**

Companion-oriented AI chatbots are diffusing rapidly among Chinese emerging adults, yet their relationship with loneliness remains unclear. Adopting a media-effects perspective that treats companion AI use as a form of media use, this exploratory study examined whether companion AI use is associated with loneliness through self-attributed offline social displacement, and whether relational-interdependent self-construal moderates this association.

**Methods:**

Using a cross-sectional survey, we administered measures of companion AI use, self-attributed offline social displacement, loneliness, and relational-interdependent self-construal to 843 Chinese emerging adults (aged 18–29 who had used a companion AI within the preceding 3 months). Because no established Chinese measures were available for the two companion-AI-specific constructs, study-specific preliminary item sets were used and their internal structure was examined with split-half EFA/CFA before the moderated mediation analyses.

**Results:**

Self-attributed offline social displacement partially mediated the relationship between companion AI use and loneliness (the indirect effect accounted for roughly one-third of the total effect). Relational-interdependent self-construal moderated the second stage of this mediation: the indirect effect through displacement was significant only among individuals with moderate-to-high interdependence and was non-significant among those with low interdependence.

**Conclusion:**

The findings are consistent with a self-reported displacement-related association in the context of generative companion AI and point to a possible boundary condition on that association tied to relational-interdependent self-construal. The results should be interpreted as preliminary and correlational because the displacement measure asked respondents to attribute reduced offline interaction to AI use and was not validated in an independent sample.

## 1 Introduction

Companion-oriented AI chatbots built on large language models are diffusing rapidly. In contrast to tool-oriented AI, which is used primarily to retrieve information or accomplish tasks, companion AI applications (e.g., Xingye, Maoxiang, Zhumengdao, Maopaoya, Feichuan) are used mainly for everyday chatting, emotional exchange, and companionship-style conversation. With their anthropomorphic style, emotionally responsive replies, and round-the-clock availability, they are becoming a new kind of social partner in the daily lives of many young people (Pentina et al., 2023; Skjuve et al., 2022). Their potential implications for users' interpersonal lives and mental health constitute a new question that warrants examination.

Emerging adulthood (approximately 18–29 years) is a developmental stage characterized by identity exploration, relational instability, and self-focus (Arnett, 2000). Individuals in this stage both rely heavily on peer relationships to construct the self and are undergoing a reorganization of their offline social networks, which makes them susceptible to loneliness (Cacioppo and Hawkley, 2009; Hawkley and Cacioppo, 2010); they are, at the same time, among the primary users of companion AI (Lai et al., 2025; Liu et al., 2026). Emerging adults are therefore a key population in which to examine the relationship between companion AI use and loneliness. Companion AI can be understood both as a conversational partner that users interact with and as an LLM-based application whose use occupies time and psychological resources. Adopting a media-effects perspective, this study treats companion AI use as a form of media use and examines whether such use is associated with the displacement of offline interaction. Rather than framing the phenomenon as addiction or problematic use, the study focuses on the underlying mechanism.

Two opposing theoretical predictions exist regarding the relationship between companion AI use and loneliness. The media-displacement hypothesis holds that the time and psychological resources invested in media use may substitute for time that would otherwise be spent on offline face-to-face interaction, thereby weakening real-world interpersonal ties and, in turn, impairing wellbeing (Kraut et al., 1998; Nie, 2001; Twenge et al., 2019). By contrast, the social-compensation or stimulation hypothesis holds that media use can strengthen existing relationships through self-disclosure, or compensate for deficits in real-world interaction (Valkenburg and Peter, 2007, 2009). Evidence for compensation derives largely from more traditional media, such as instant messaging and social-networking sites, and from adolescent samples (Kraut et al., 2002; Nowland et al., 2018); whether these conclusions generalize to generative companion AI remains to be tested. Accordingly, this study does not claim to adjudicate displacement against compensation. Instead, it asks whether heavier companion-oriented AI use is associated with respondents' own attribution that AI use has displaced some offline interaction, and whether that self-attributed displacement is associated with loneliness.

The anthropomorphism, emotional responsiveness, and around-the-clock availability of companion AI make it a potential functional substitute for offline in-person interaction. Higher usage may therefore be associated with a stronger perception that AI chatting has taken time away from offline face-to-face interaction. The present study focuses on such self-attributed, behavioral-and-temporal substitution, rather than on objectively measured reductions in social time. We therefore propose Hypothesis 1 (H1): companion AI use will be positively associated with self-attributed offline social displacement.

Offline face-to-face interaction is a central route to satisfying the need to belong; belongingness is met through frequent positive interaction embedded in stable, mutually caring, ongoing relationships (Baumeister and Leary, 1995; Mellor et al., 2008). When individuals report that offline interaction has been displaced by AI, the ongoing-relationship component of belongingness may be undermined, and loneliness may be higher. Although AI can provide frequent, immediate responses, it lacks genuine reciprocity and social validation from a real person; it thus does not constitute a stable, mutually caring relationship and cannot, on balance, substitute for what offline interaction supplies to belongingness. We therefore propose Hypothesis 2 (H2): self-attributed offline social displacement will be positively associated with loneliness; and Hypothesis 3 (H3): companion AI use will be indirectly and positively associated with loneliness through self-attributed offline social displacement.

Self-construal theory distinguishes independent from interdependent self-construal and holds that the relative salience of these orientations is shaped by cultural context: interdependent self-construal, in which the self is defined through connectedness to others, is more prevalent and normative in East Asian and Chinese cultural contexts than in Western ones (Markus and Kitayama, 1991). Relational-interdependent self-construal refers more specifically to the tendency to define the self in terms of close relationships with others (Cross et al., 2000). Although it is a culturally shaped orientation, it also varies across individuals within a given culture, and it is this within-culture variation that the present study examines. For individuals high in relational interdependence, who anchor the self more firmly in real-world close relationships, such relationships are less substitutable for belongingness and the self; self-attributed displacement should therefore be more strongly associated with loneliness. We therefore propose Hypothesis 4 (H4): relational-interdependent self-construal moderates the relationship between self-attributed social displacement and loneliness, such that the positive association between displacement and loneliness is stronger at higher levels of interdependence; and Hypothesis 5 (H5): the indirect association between companion AI use and loneliness through self-attributed social displacement strengthens as relational interdependence increases.

Taken together, this study specifies and tests a moderated mediation model (see Figure 1) among Chinese emerging adults, in order to examine whether the observed associations are consistent with a displacement-related process in the context of generative companion AI, and to characterize how cultural self-construal moderates this pathway.

**Figure 1:**
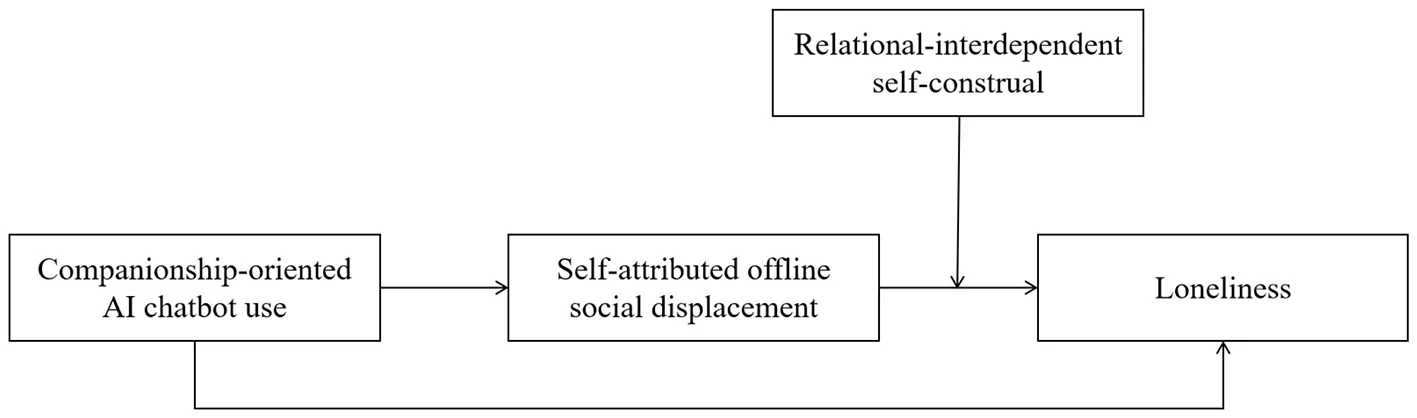
Conceptual model.

## 2 Methods

### 2.1 Participants and procedure

This study used a cross-sectional survey, recruiting Chinese emerging adults through the online survey platform Wenjuanxing (Sojump). Upon completing the questionnaire, participants received a small cash incentive. Eligibility screening required participants to be aged 18–29 and to have used, within the preceding 3 months, a companion AI whose primary purpose is everyday chatting, emotional exchange, or companionship-style conversation (e.g., Xingye, Maoxiang, Zhumengdao, Maopaoya, Feichuan); the questionnaire terminated automatically for those who did not qualify. Informed consent was presented before the questionnaire began, and only participants who confirmed their consent proceeded; responses were anonymous throughout.

A total of 940 questionnaires were collected. Invalid responses were removed: respondents who failed two or more of the three attention-check items, or whose total completion time was shorter than one-third of the sample median, were excluded. On this basis, 97 responses were removed, yielding 843 valid questionnaires (valid response rate = 89.68%). The measurement-model and discriminant-validity analyses were based on the full sample (*N* = 843); the exploratory and confirmatory factor analyses of the two study-specific preliminary item sets used a split-half design (EFA *n* approximately 421; CFA *n* = 422). The primary moderated mediation analysis was based on complete cases after all control variables were included (*N* = 783); listwise deletion removed 60 cases, with missing data concentrated in offline social frequency, daily AI-use duration, and length of AI-use history. The sample was predominantly female (55.5%) and aged 18–22 (53.0%), and most participants held a bachelor's degree (60.6%); full demographic and usage characteristics are reported in Supplementary Table 1.

This study received approval from the Ethics Committee of Yunnan Normal University (Approval No. YNNU-EC-2025-0173).

### 2.2 Measures

To reduce common method bias procedurally, each variable block used a different response anchor: companion AI use was scored on a 5-point agreement scale, relational-interdependent self-construal on a 7-point agreement scale, self-attributed offline social displacement on a 5-point agreement scale, and loneliness on a 4-point frequency scale; the order in which the blocks were presented was also arranged deliberately, and one attention-check item was embedded within each of the main scale blocks (three in total).

#### 2.2.1 Companion AI use

No established Chinese scale currently measures “companion-oriented AI use.” Existing instruments focus mainly on dependence or problematic use, which does not match the neutral construct of use in this study. We therefore constructed a 7-item study-specific preliminary item set for this purpose. Sample items include “I often use AI chatbots for chatting and conversation” and “Beyond looking up information or getting things done, I also use AI simply for chatting and companionship.” Items were scored on a 5-point Likert scale (1 = strongly disagree, 5 = strongly agree), with higher scores indicating greater companion AI use. Internal consistency was good in this sample (alpha = 0.814, omega = 0.816).

#### 2.2.2 Self-attributed offline social displacement

Self-attributed offline social displacement refers to an individual's subjective perception that using companion AI has substituted for, or displaced, offline face-to-face interaction with real people. Because no established scale directly measuring this construct is currently available, we constructed a 7-item study-specific preliminary item set (e.g., “Some of the time I used to spend with friends or family in person, I now spend chatting with AI;” “Because I often chat with AI, I have reduced how often I arrange to meet people offline”; one item is reverse-scored). The items intentionally capture respondents' attribution that AI use is linked to reduced offline interaction; accordingly, this variable should not be interpreted as an objective behavioral measure of displacement. Items were scored on a 5-point Likert scale (1 = strongly disagree, 5 = strongly agree), with higher scores indicating stronger self-attributed displacement. Internal consistency was good in this sample (alpha = 0.813, omega = 0.816).

#### 2.2.3 Loneliness

Loneliness was measured with the 6-item UCLA Loneliness Scale (ULS-6; Neto, 1992; Xiao and Du, 2023). The ULS-6 is a short form of the UCLA Loneliness Scale (Russell, 1996), and its Chinese version has shown good reliability and validity in a Chinese adult sample (Xiao and Du, 2023). The scale comprises 6 items (e.g., “I often feel that I lack companionship;” “I often feel that I am a lonely person”), scored on a 4-point Likert scale (1 = never, 4 = always), with higher scores indicating greater loneliness. Internal consistency was good in this sample (α = 0.816, ω = 0.816).

#### 2.2.4 Relational-interdependent self-construal

Relational-interdependent self-construal was measured with the Chinese version of the Relational-Interdependent Self-Construal Scale (Huang and Bi, 2012; for the original English scale, see Cross et al., 2000). We chose relational interdependence rather than general interdependence for two reasons. First, it focuses specifically on “defining the self through close interpersonal relationships.” This mechanism concerns how real-world relationships are displaced by a medium, and relational interdependence maps onto it more directly. Second, it avoids the long-standing factorial-validity disputes surrounding general self-construal instruments (Cross et al., 2011). The scale comprises 9 items with a unidimensional structure, scored on a 7-point Likert scale (1 = strongly disagree, 7 = strongly agree), with higher scores indicating greater relational-interdependent self-construal. Internal consistency was good in this sample (α = 0.866, ω = 0.866).

#### 2.2.5 Control variables

The following control variables were included: age (in categories, e.g., 18–22, 23–25, 26–29 years), education (high school or below; junior college; bachelor's; master's or above), living arrangement (with family / with roommates or a partner / living alone), weekly frequency of offline face-to-face contact with friends or family, average daily duration of companion AI use, and length of companion AI use history. (Gender was not entered as a control in the main model, because its coding included a small number of “other / prefer not to say” responses, dichotomizing it would lose cases, and it was only weakly related to the core variables.)

### 2.3 Confirmatory factor analysis

Before the main analyses, we conducted confirmatory factor analyses of the four constructs to examine the measurement model. For the two study-specific preliminary item sets (companion AI use and self-attributed offline social displacement), we used a split-half strategy: the sample was randomly divided into halves, one used for exploratory factor analysis and the other (*n* = 422) for confirmatory factor analysis. This provided preliminary evidence of internal structure while avoiding EFA and CFA in exactly the same subsample, but it should not be taken as full independent validation of the measures. Each item set fit the measurement model well in the validation half-sample, and the four-factor measurement model showed acceptable overall fit.

### 2.4 Data analysis

Statistical analyses proceeded in two stages. Measurement-model and reliability/validity analyses were conducted with the lavaan package in R (Rosseel, 2012): confirmatory factor analyses used robust maximum-likelihood estimation, and CFI, TLI, RMSEA, and SRMR were reported; the two study-specific item sets used a split-half strategy, with one half for exploratory factor analysis and the other (*n* = 422) for confirmatory factor analysis; McDonald's omega was computed from the standardized factor loadings and residual variances of the confirmatory model, and Cronbach's alpha was computed together with descriptive statistics and correlations. Discriminant validity was assessed with the heterotrait-monotrait ratio (HTMT) (Henseler et al., 2015) and was further examined by comparing, for the target constructs, a two-factor model against a combined one-factor model. Common method bias was addressed primarily through procedural remedies, supplemented by Harman's single-factor test and single–vs. multi-factor model comparisons (Podsakoff et al., 2003). Mediation and moderated mediation analyses were conducted in SPSS with the PROCESS macro (Models 4 and 14; Hayes, 2022). Path coefficients were estimated by regression, with variables mean-centered before testing the interaction term. Indirect effects, the index of moderated mediation, and conditional indirect effects at different levels of the moderator were obtained using bias-corrected bootstrapping (5,000 resamples); a 95% confidence interval excluding zero indicated significance. To address concerns about the covariate set and the interpretation of the X-to-M path, we additionally ran sensitivity models excluding daily AI-use duration, excluding offline social frequency, and treating offline social frequency as an external criterion.

## 3 Results

### 3.1 Common method bias

Harman's single-factor test indicated that the first factor accounted for 19.26% of the variance, which did not constitute evidence of substantial bias. We further compared a single-factor with a four-factor model in the full sample (*N* = 843): the single-factor model fit poorly (CFI = 0.486, TLI = 0.447, RMSEA = 0.109, SRMR = 0.126), whereas the four-factor model fit well (CFI = 0.984, TLI = 0.982, RMSEA = 0.019, SRMR = 0.028), indicating that common method bias was unlikely to account for all of the observed relationships.

### 3.2 Descriptive statistics and correlations

The cleaned valid sample was N = 843. Reliability was good for all four scales: companion AI use (α = 0.814, ω = 0.816), relational-interdependent self-construal (α = 0.866, ω = 0.866), self-attributed offline social displacement (α = 0.813, ω = 0.816), and loneliness (α = 0.816, ω = 0.816). Descriptive statistics and the correlation matrix are presented in Table 1. Correlational analysis showed that companion AI use, self-attributed social displacement, loneliness, and relational-interdependent self-construal were pairwise positively correlated.

**Table 1:** Descriptive statistics and correlation matrix (*N* = 843).

Variable	M	SD	1	2	3	4	5	6	7
1 Companion AI use	3.154	0.951	(0.81)						
2 Self-attributed displacement	3.138	0.948	0.442^***^	(0.81)					
3 Loneliness	2.519	0.798	0.351^***^	0.364^***^	(0.82)				
4 Relational interdependence	5.173	1.282	0.130^***^	0.209^***^	0.135^***^	(0.87)			
5 Gender	0.435	0.496	−0.025	−0.019	−0.073^*^	0.024	—		
6 Age	1.668	0.787	0.071^*^	0.012	0.033	0.017	0.063	—	
7 Offline social frequency	3.224	1.210	−0.112^**^	−0.186^***^	−0.090^**^	−0.084^*^	0.077^*^	0.013	—
8 Daily AI use duration	2.608	1.122	0.495^***^	0.253^***^	0.204^***^	0.091^**^	−0.038	0.052	−0.076^*^

Unless otherwise stated, descriptive statistics were based on the cleaned sample (*N* = 843). Cronbach's α is shown in parentheses on the diagonal. Gender coded male = 1, female = 0; other control variables coded in category order. Gender statistics were based on *n* = 829, excluding 14 participants who selected “other/prefer not to disclose;” offline social frequency, daily AI use duration, and length of AI use history had 22, 21, and 20 missing values, respectively. Education, living arrangement, and length of AI use history were entered as categorical covariates in the regression models and are not shown in this matrix (see Supplementary Table 1 for their distributions). ^*^p < 0.05, ^**^p < 0.01, ^***^p < 0.001.

### 3.3 Measurement model and discriminant validity

For the two study-specific preliminary item sets, exploratory factor analysis in the first half-sample supported a unidimensional structure: sampling adequacy was acceptable (companion AI use, KMO = 0.882; self-attributed displacement, KMO = 0.864), and each item set showed a single dominant factor, with the first two eigenvalues clearly separated (companion AI use: 3.219 and 0.798; self-attributed displacement: 3.197 and 0.876). Confirmatory factor analyses of each item set indicated sound structure (see Table 2, Panel A); in the split-half validation sample (*n* = 422), the four-factor model fit well overall (CFI = 0.979, TLI = 0.977, RMSEA = 0.022, SRMR = 0.036). Regarding discriminant validity, all pairwise HTMT values were below 0.85 (see Table 2, Panel D); for self-attributed social displacement and loneliness, HTMT was 0.447, with a bootstrap 95% CI of [0.371, 0.523], the upper bound of which did not cross the threshold. We further compared, for these two constructs, a two-factor model against a combined one-factor model (see Table 2, Panel C): the two-factor model fit well (CFI = 0.992, TLI = 0.990, RMSEA = 0.022, SRMR = 0.021), whereas the combined one-factor model fit markedly worse (CFI = 0.712, TLI = 0.654, RMSEA = 0.127, SRMR = 0.099), and the two models differed significantly, chi-square difference (1) = 856.79, *p* < 0.001. These results indicate that self-attributed offline social displacement and loneliness are distinguishable constructs, although they do not address the possible shared item content between companion AI use and self-attributed displacement.

**Table 2:** Confirmatory factor analysis and model comparisons.

Model	χ^2^	Df	*P*	CFI	TLI	RMSEA	SRMR
Panel A. Individual scales and the four-factor model (split-half validation sample, *n* = 422)							
Companion AI use	10.509	14	0.724	1.000	1.006	0.000	0.017
Self-attributed displacement	19.706	14	0.140	0.993	0.990	0.031	0.021
Loneliness	11.182	9	0.263	0.997	0.994	0.024	0.019
Relational interdependence	53.902	27	0.002	0.976	0.967	0.049	0.029
Four-factor model	446.366	371	0.004	0.979	0.977	0.022	0.036
Panel B. Common method bias test (full sample, *N* = 843)							
Single-factor model	4,144.828	377	< 0.001	0.486	0.447	0.109	0.122
Four-factor model	488.620	371	< 0.001	0.984	0.982	0.019	0.028
Panel C. One–vs. two-factor comparison for displacement and loneliness (full sample, *N* = 843)							
M–Y two-factor	89.979	64	0.018	0.992	0.990	0.022	0.021
M + Y one-factor	946.768	65	< 0.001	0.712	0.654	0.127	0.099
Variable	1		2		3		4
Panel D. Discriminant validity (HTMT, *N* = 843)							
1 Companion AI use	—						
2 Self-attributed displacement	0.543		—				
3 Loneliness	0.430		0.447		—		
4 Relational interdependence	0.156		0.249		0.161		—

CFA used robust maximum-likelihood estimation in R (lavaan); Δχ^2^ (1) = 856.789, *p* < 0.001. HTMT values were computed from the item correlation matrix and were all below 0.85.

### 3.4 Mediation analysis

The mediation and moderated mediation analyses were based on complete cases after all control variables were included (*N* = 783). Regression results showed that companion AI use was positively associated with self-attributed offline social displacement (*a* = 0.398, SE = 0.037, *t* = 10.73, *p* < 0.001), and that self-attributed offline social displacement was positively associated with loneliness (*b* = 0.213, SE =0.031, *t* = 6.96, *p* < 0.001); the indirect effect through displacement was significant [a x b = 0.085, 95% BC CI (0.056, 0.119)]. The total effect was significant (*c* = 0.254, SE = 0.033, *t* = 7.79, *p* < 0.001), and the direct effect remained significant after the mediator was included (c' = 0.169, SE = 0.034, *t* = 4.99, *p* < 0.001); the indirect effect accounted for approximately 33.4% of the total effect, and the total and direct effects were both positive, indicating partial mediation in associational terms (see Table 3). Multicollinearity was not a concern in the regression models. All variance inflation factors were < = 1.54. Daily AI-use duration was the covariate most closely related to the predictor, and its VIF was 1.34.

**Table 3:** Mediation effect of self-attributed social displacement (*N* = 783).

Path/effect	b	SE	*t*	*p*	95% CI
a: AI use → displacement	0.398	0.037	10.73	< 0.001	[0.325,0.471]
b: displacement → loneliness	0.213	0.031	6.96	< 0.001	[0.153,0.273]
c: total effect	0.254	0.033	7.79	< 0.001	[0.190,0.318]
c′: direct effect	0.169	0.034	4.99	< 0.001	[0.103,0.236]
a × b: indirect effect	0.085	—	—	—	[0.056,0.119]

Six covariates were controlled (age, education, living arrangement, offline social frequency, daily AI use duration, and length of AI use history); path coefficients are SPSS regression output, and the indirect effect is based on 5,000 PROCESS bootstrap resamples (bias-corrected CI).

### 3.5 Moderated mediation analysis

The moderated mediation analysis showed that the interaction between self-attributed social displacement and relational-interdependent self-construal was significantly and positively associated with loneliness (M x W: b = 0.102, SE = 0.019, *t* = 5.35, *p* < 0.001); the main effect of relational interdependence was small but significant (W: b = 0.042, SE = 0.021, *t* = 2.02, *p* = 0.043), and companion AI use was also significantly and positively associated with loneliness (X: b = 0.163, SE = 0.033, *t* = 4.89, *p* < 0.001). The index of moderated mediation was significant [index = 0.041, 95% BC CI (0.025, 0.059)]. Simple-slope tests indicated that the positive association between self-attributed social displacement and loneliness strengthened as relational interdependence increased: at low interdependence (−1 SD), b = 0.080 (*p* = 0.040); at the mean, b = 0.211 (*p* < 0.001); at high interdependence (+1 SD), b = 0.343 (*p* < 0.001). Conditional indirect effects showed that the indirect effect was significant at moderate [0.084, 95% BC CI (0.056, 0.117)] and high [0.137, 95% BC CI (0.098, 0.179)] levels of interdependence, but not at the low level [0.032, 95% BC CI (−0.001, 0.067)]. Overall, the indirect association from companion AI use to loneliness through self-attributed social displacement held mainly among individuals with moderate-to-high relational interdependence (see Table 4).

**Table 4:** Moderated mediation analysis (*N* = 783).

Panel A. Regression model (outcome: loneliness)					
Predictor	b	SE	*t*	*p*	95% CI
AI use (X)	0.163	0.033	4.89	< 0.001	[0.097,0.228]
Displacement (M)	0.211	0.031	6.92	< 0.001	[0.151,0.271]
Relational interdependence (W)	0.042	0.021	2.02	0.043	[0.001,0.082]
M × W	0.102	0.019	5.35	< 0.001	[0.065,0.140]
Panel B. Conditional indirect effects at different levels of W					
Level of W	Indirect effect		95% BC CI	Significant	
−1 SD	0.032		[−0.001,0.067]	No	
Mean	0.084		[0.056,0.117]	Yes	
+1 SD	0.137		[0.098,0.179]	Yes	
Index of moderated mediation	0.041		[0.025,0.059]	Yes	
Panel C. Simple slopes (displacement→loneliness)					
Level of W	b	SE	*T*	*p*	95% CI
−1 SD	0.080	0.039	2.05	0.040	[0.004,0.156]
Mean	0.211	0.031	6.92	< 0.001	[0.151,0.271]
+1 SD	0.343	0.040	8.64	< 0.001	[0.265,0.421]

Panel A path coefficients are SPSS regression output (variables mean-centered); the index of moderated mediation and the conditional indirect effects are based on 5,000 PROCESS bootstrap resamples (bias-corrected CI). At low interdependence, the simple slope was nominally significant, but the bias-corrected CI of the conditional indirect effect included zero, so the effect was judged non-significant at the mediational level. Because the M → Y relationship is qualified by the M × W interaction in Model 14, the coefficient of M reported here is the conditional effect at the mean of W and therefore differs slightly from the unconditional b path in Table 3 (Model 4; b = 0.213).

The simple-slope plot (Figure 2) shows two crossing slope lines, with the crossover at a raw displacement value of approximately 2.71 (within the +/−1 SD range): at the low-displacement end, the estimated loneliness of highly interdependent individuals was slightly lower than that of individuals low in interdependence (2.226 vs. 2.370), whereas at the high-displacement end it was markedly higher (2.879 vs. 2.521). This pattern suggests that relational interdependence may operate as a boundary condition: it appears protective when self-attributed displacement is low, but is associated with greater loneliness when self-attributed displacement is high. Sensitivity analyses produced the same substantive pattern (Supplementary Table 2). When daily AI-use duration was excluded from the covariates, the X-to-M path remained positive (b = 0.418, *p* < 0.001), the M-to-Y path remained positive (b = 0.215, *p* < 0.001), and the M x W interaction remained significant (b = 0.101, *p* < 0.001). When offline social frequency was excluded, the corresponding coefficients also remained positive and significant (X-to-M: b = 0.412, *p* < 0.001; M-to-Y: b = 0.214, *p* < 0.001; M x W: b = 0.102, *p* < 0.001). In an external-criterion check (Supplementary Table 3), companion AI use was weakly but significantly associated with lower offline social frequency (b = −0.119, *p* = 0.023), but this association was no longer significant after self-attributed displacement was included (b = −0.027, *p* = 0.628), whereas self-attributed displacement remained negatively associated with offline frequency (b = −0.224, *p* < 0.001). These results support interpreting the mediator as respondents' self-attributed displacement experience rather than as direct evidence of objective behavioral displacement.

**Figure 2:**
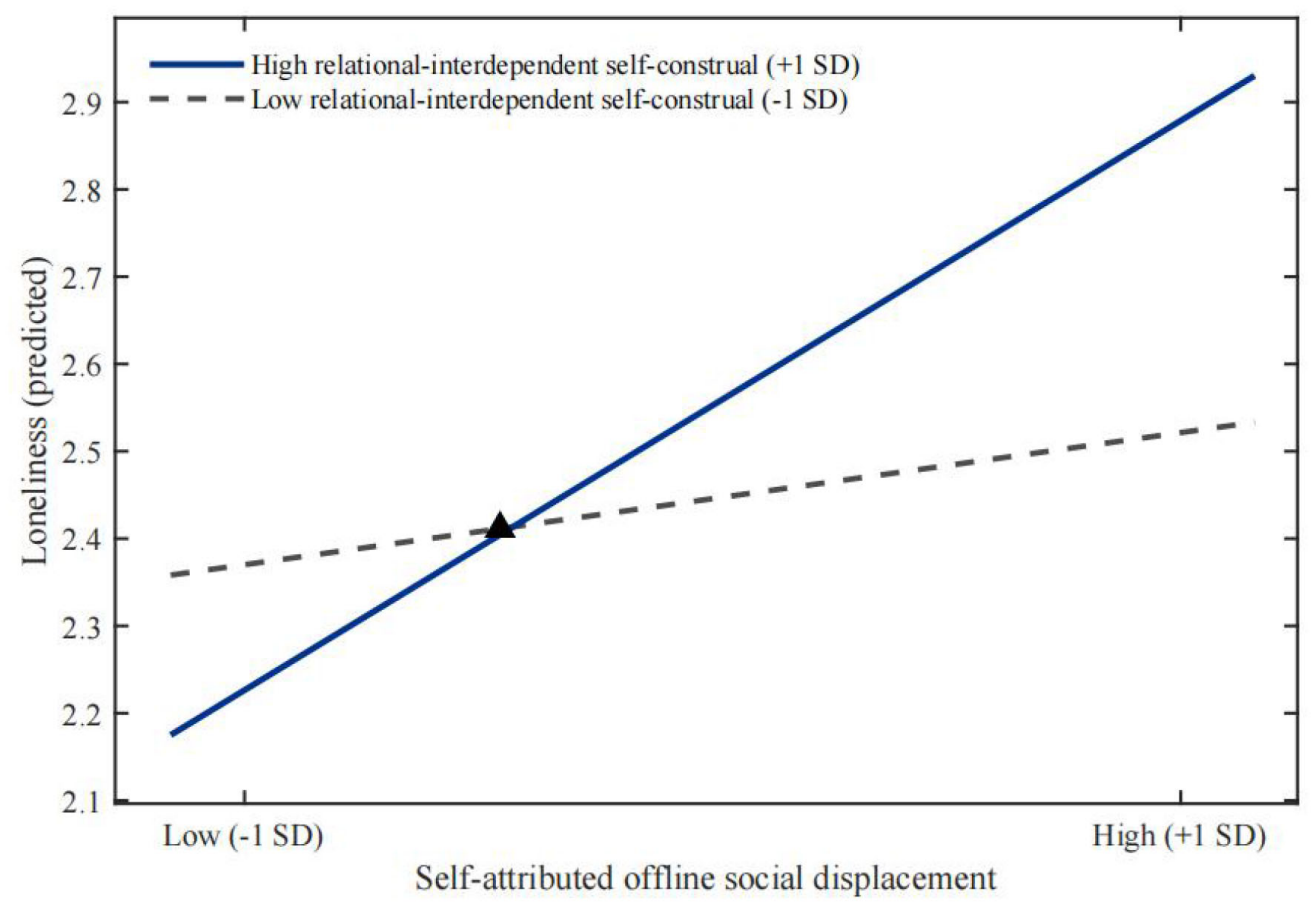
Simple-slope plot of displacement predicting loneliness at different levels of relational interdependence.

## 4 Discussion

This study examined the association between companion AI use and loneliness, and the role of self-attributed offline social displacement, among Chinese emerging adults. The results indicate that companion AI use was positively associated with loneliness through self-attributed offline social displacement, constituting partial mediation in associational terms, and that this indirect association was moderated by relational-interdependent self-construal, holding mainly when an individual's relational interdependence reached a moderate or higher level. We discuss four issues in turn: the discriminant validity of self-attributed social displacement and loneliness, the displacement-related association, the moderation by relational interdependence and its boundary, and the implications and limitations of the study.

### 4.1 Discriminant validity of self-attributed social displacement and loneliness

A key premise of this study is that self-attributed offline social displacement and loneliness are two distinguishable constructs rather than two names for the same experience. The data provide evidence for this distinction: HTMT between the two was 0.447 (upper bound of the 95% CI = 0.523), well below 0.85 (Henseler et al., 2015), and the combined one-factor model fit significantly worse than the two-factor model (chi-square difference (1) = 856.79, *p* < 0.001). This supports the separability of self-attributed displacement and loneliness. At the same time, this evidence does not eliminate a different concern: some displacement items explicitly refer to AI use as the attributed cause of reduced offline interaction. Therefore, the X-to-M association should be interpreted cautiously as an association between companion AI use and AI-attributed displacement reports, not as proof that AI use objectively displaced offline interaction.

### 4.2 The mediating mechanism of displacement and partial mediation

This study provides preliminary evidence consistent with extending the media-displacement hypothesis from traditional media to generative companion AI: companion AI use was associated with individuals' subjective attribution that offline interaction with real people had been displaced, and this self-attributed displacement was in turn associated with higher loneliness. This result is consistent with belongingness theory (Baumeister and Leary, 1995): in theoretical terms, when offline interaction with real people is perceived as displaced, the ongoing-relationship component on which belongingness depends may be undermined, and loneliness may be correspondingly higher.

Notably, the present result is partial rather than full mediation: the total and direct effects were both positive, indicating that self-attributed displacement is only one of the paths linking companion AI use to loneliness and that other, non-mediated associations remain. This is not inconsistent with recent evidence that companion AI can relieve momentary loneliness (De Freitas et al., 2026; Maples et al., 2024): companion AI may involve both immediate comfort and longer-term loneliness consequences via self-attributed social displacement, the two coexisting along different paths and time scales, with the net outcome further depending on moderators such as social connectedness and prior loneliness (Nakagomi et al., 2026). In other words, whether companion AI is beneficial or harmful is not one-directional; it appears to depend partly on whether users perceive that AI use has replaced offline interaction with real people.

### 4.3 The moderating role of relational interdependence and its boundary

The moderation by relational-interdependent self-construal appeared to take the form of a boundary condition rather than simple amplification. The simple-slope plot shows two crossing lines. When self-attributed displacement was low, highly interdependent individuals reported slightly lower loneliness, which is consistent with a protective role. Once displacement was perceived, the pattern reversed. Because their belongingness is more deeply anchored in real-world close relationships, perceived substitution of those relationships may be more strongly associated with loneliness.

The result at the low-interdependence end is particularly noteworthy: for individuals low in relational interdependence, the indirect association through displacement was non-significant. One possible explnation is that their self-worth and belongingness depend less on real-world close relationships. As a result, when they report AI-attributed displacement, the threat to belongingness may be weaker and less readily translated into loneliness. This suggests that relational interdependence may bear on whether the displacement-related association is engaged at all. Because relational-interdependent self-construal is a culturally shaped dimension of the self, we characterize this as an individual-difference boundary condition rooted in a culturally shaped construct rather than as a purely idiosyncratic one. This pattern echoes the claim in self-construal theory concerning the centrality of relationships to self-definition (Cross et al., 2000), and accords with evidence that self-construal moderates subjective wellbeing in emerging adulthood (Hatano et al., 2024).

### 4.4 Implications and limitations

This study has certain theoretical and practical implications. Theoretically, by focusing on a displacement-related association rather than only the total association between AI use and loneliness, it extends media-effects research to generative companion AI and suggests how cultural self-construal may shape the psychological correlates of media use. Practically, loneliness risk appears to be related to users' perception that companion AI has replaced offline interaction with real people, rather than to use it self alone; interventions might therefore concentrate on preserving real-world interaction while allowing moderate AI use. These recommendations should be read cautiously and not extended beyond what cross-sectional data can support. This overall picture is consistent with a growing body of recent work, some reporting benefits and some reporting risks (Herbener and Damholdt, 2025; Jain et al., 2026; Lai et al., 2025; Marriott and Pitardi, 2024; Zhang et al., 2025).

This study also has several limitations. First, its cross-sectional design cannot establish causal or temporal relations among the variables; all reported effects are associations, and the direction X-to-M-to-Y is set by theory rather than tested by the data. Second, companion AI use and self-attributed offline social displacement were measured with study-specific preliminary item sets. Although reliability and internal-structure evidence were acceptable, the validity evidence comes from a single cross-sectional sample and requires independent validation across samples and contexts. Third, the displacement item set explicitly asked respondents to attribute reduced offline interaction to AI use. This is appropriate for measuring self-attributed displacement, but it means that the X-to-M path may be partly strengthened by shared item content and should not be interpreted as evidence that heavier AI use objectively caused offline social displacement. Fourth, because the data came from a single cultural context, the moderating role of relational-interdependent self-construal reflects within-culture individual variation; claims about genuine between-culture differences would require cross-cultural comparative designs. Fifth, displacement was measured as a subjective perception and did not incorporate behavioral indicators such as objective social time; future work could combine behavioral data with multi-method measurement.

## 5 Conclusion

Among Chinese emerging adults, companion AI use was positively associated with loneliness through self-attributed offline social displacement, and this indirect association held mainly among individuals with moderate-to-high relational interdependence. The findings suggest that loneliness-related risks may be tied less to companion AI use *per se* than to users' perception that such use has replaced offline interaction with real people. Because the measures of companion AI use and self-attributed displacement were preliminary and the data were cross-sectional, the findings should be regarded as exploratory evidence that motivates independent measurement validation and longitudinal or experimental tests.

## Data Availability

The raw data supporting the conclusions of this article will be made available by the authors, without undue reservation.
