# Supplementary material for: Companion-oriented AI chatbot use and loneliness among Chinese emerging adults: the mediating role of self-attributed offline social displacement and the moderating role of relational-interdependent self-construal
Source: Front Psychol. 2026 Oct 22;17:1933530. doi: 10.3389/fpsyg.2026.1933530 (PMC13735586; doi:10.3389/fpsyg.2026.1933530)
Supplement: Supplementary file 1 [file Supplementary_File_1.docx]

# Supplementary Material

**Supplementary Table S1. Participants' demographic and usage characteristics (N = 843).**

| Variable | Category | n | % |
| --- | --- | --- | --- |
| Gender | Female | 468 | 55.5 |
|  | Male | 361 | 42.8 |
|  | Other / undisclosed | 14 | 1.7 |
| Age | 18–22 years | 447 | 53.0 |
|  | 23–25 years | 229 | 27.2 |
|  | 26–29 years | 167 | 19.8 |
| Education | High school or below | 45 | 5.3 |
|  | Junior college | 169 | 20.0 |
|  | Bachelor's | 511 | 60.6 |
|  | Master's or above | 118 | 14.0 |
| Living arrangement | With family | 239 | 28.4 |
|  | With roommates / partner | 385 | 45.7 |
|  | Living alone | 219 | 26.0 |
| Offline social frequency | Almost none | 80 | 9.5 |
|  | A few times per month | 162 | 19.2 |
|  | About once per week | 200 | 23.7 |
|  | Several times per week | 252 | 29.9 |
|  | Almost daily | 127 | 15.1 |
|  | Missing | 22 | 2.6 |
| Daily AI use duration | < 10 min | 109 | 12.9 |
|  | 10–30 min | 347 | 41.2 |
|  | 30–60 min | 190 | 22.5 |
|  | 1–2 h | 109 | 12.9 |
|  | > 2 h | 67 | 7.9 |
|  | Missing | 21 | 2.5 |
| Length of AI use history | < 1 month | 134 | 15.9 |
|  | 1–6 months | 346 | 41.0 |
|  | 6–12 months | 217 | 25.7 |
|  | > 1 year | 126 | 14.9 |
|  | Missing | 20 | 2.4 |

*Note.* Percentages are of the full valid sample (N = 843) and may not sum to 100 due to rounding. Age, education, living arrangement, offline social frequency, daily AI use duration, and length of AI use history were entered as ordinal/categorical control variables in the regression models.

**Supplementary Table S2. Sensitivity analyses excluding selected covariates (complete-case N = 783).**

| Model | Coefficient | b | SE | t | p |
| --- | --- | --- | --- | --- | --- |
| Excluding daily AI-use duration | X to M | .418 | .033 | 12.88 | < .001 |
| Excluding daily AI-use duration | M to Y | .215 | .031 | 7.01 | < .001 |
| Excluding daily AI-use duration | M x W to Y | .101 | .019 | 5.27 | < .001 |
| Excluding offline social frequency | X to M | .412 | .038 | 10.99 | < .001 |
| Excluding offline social frequency | M to Y | .214 | .030 | 7.08 | < .001 |
| Excluding offline social frequency | M x W to Y | .102 | .019 | 5.36 | < .001 |

*Note.* X = companion AI use; M = self-attributed offline social displacement; W = relational-interdependent self-construal. All models retained age, education, living arrangement, and AI-use history as covariates; the first set also retained offline social frequency, and the second set also retained daily AI-use duration. Coefficients are unstandardized OLS estimates from the rerun analysis.

**Supplementary Table S3. Offline social frequency as an external criterion (complete-case N = 783).**

| Model | Predictor | b | SE | t | p |
| --- | --- | --- | --- | --- | --- |
| Offline frequency regressed on X and covariates | X | -.119 | .052 | -2.28 | .023 |
| Offline frequency regressed on X, M, and covariates | X | -.027 | .056 | -0.48 | .628 |
| Offline frequency regressed on X, M, and covariates | M | -.224 | .050 | -4.52 | < .001 |

*Note.* Offline social frequency was coded so that higher values indicated more frequent face-to-face contact with friends or family. Covariates were age, education, living arrangement, daily AI-use duration, and AI-use history. These results support interpreting the mediator as self-attributed displacement rather than as direct evidence of objective behavioral displacement.
